# Diagnosis of Human Axillary Osmidrosis by Genotyping of the Human* ABCC11* Gene: Clinical Practice and Basic Scientific Evidence

**DOI:** 10.1155/2016/7670483

**Published:** 2016-02-23

**Authors:** Yu Toyoda, Tsuneaki Gomi, Hiroshi Nakagawa, Makoto Nagakura, Toshihisa Ishikawa

**Affiliations:** ^1^Department of Pharmacy, The University of Tokyo Hospital, 7-3-1 Hongo, Bunkyo-ku, Tokyo 113-8655, Japan; ^2^Gomi Clinic, 1-10-12 Hyakunin-cho, Shinjyuku-ku, Tokyo 169-0073, Japan; ^3^Department of Applied Biological Chemistry, Graduate School of Bioscience and Biotechnology, Chubu University, 1200 Matsumoto-cho, Kasugai 487-8501, Japan; ^4^BioTec Co., Ltd., 2-29-4 Yushima, Bunkyo-ku, Tokyo 113-0034, Japan; ^5^RIKEN Center for Life Science Technology, 1-7-22 Suehiro-cho, Tsurumi-ku, Yokohama 230-0045, Japan; ^6^NGO Personalized Medicine & Healthcare, Yokohama 226-0016, Japan

## Abstract

The importance of personalized medicine and healthcare is becoming increasingly recognized. Genetic polymorphisms associated with potential risks of various human genetic diseases as well as drug-induced adverse reactions have recently been well studied, and their underlying molecular mechanisms are being uncovered by functional genomics as well as genome-wide association studies. Knowledge of certain genetic polymorphisms is clinically important for our understanding of interindividual differences in drug response and/or disease risk. As such evidence accumulates, new clinical applications and practices are needed. In this context, the development of new technologies for simple, fast, accurate, and cost-effective genotyping is imperative. Here, we describe a simple isothermal genotyping method capable of detecting single nucleotide polymorphisms (SNPs) in the human ATP-binding cassette (ABC) transporter* ABCC11* gene and its application to the clinical diagnosis of axillary osmidrosis. We have recently reported that axillary osmidrosis is linked with one SNP 538G>A in the* ABCC11* gene. Our molecular biological and biochemical studies have revealed that this SNP greatly affects the protein expression level and the function of ABCC11. In this review, we highlight the clinical relevance and importance of this diagnostic strategy in axillary osmidrosis therapy.

## 1. Introduction

Body odor production is a physiological characteristic of animals, including humans. In humans, however, strong or specific body odors are sometimes considered to be unpleasant. Axillary osmidrosis is known as a phenomenon characterized by strong body odor and profuse sweating from the armpits resulting from an excessive secretion of the body's metabolites from well-developed apocrine glands in the axillae.

Recently, we have reported that axillary osmidrosis is linked with one single nucleotide polymorphism (SNP) 538G>A in exon 4 of the human* ABCC11* gene, a member of the human ABC transporter gene family. This SNP 538G>A (rs17822931) in the* ABCC11* gene is a nonsynonymous polymorphism that alters one amino acid at position 180 from Glycine (Gly180) to Arginine (Arg180) in the ABCC11 protein. As discussed later, this amino acid substitution enhances proteasomal degradation of the SNP variant (Arg180) of* de novo* synthesized ABCC11 protein and disruption of its transport function [1]. Human subjects carrying homozygous alleles of 538G/G or heterozygous alleles of 538G/A have a higher risk of axillary osmidrosis, whereas those who are carrying the homozygous allele of 538A/A have no risk [1–3]. This association has also been confirmed within various ethnic groups [4, 5].

The* ABCC11* gene is located on human chromosome 16q12.1 [6–8]. ABCC11, also known as multidrug resistance-associated protein 8 (MRP8), is a full ABC transporter with a total of 12 putative transmembrane domains and two ATP-binding cassettes [9]. The predicted amino acid sequence of ABCC11 shows high similarity to those of ABCC4 and ABCC5 among the C family of human ABC transporters [6]. Functional assays have demonstrated that ABCC11 WT (Gly180) is able to transport a variety of lipophilic organic anions including cyclic nucleotides, glutathione conjugates such as leukotriene C_4_ (LTC_4_) and S-(2,4-dinitrophenyl)-glutathione (DNP-SG), steroid sulfates such as dehydroepiandrosterone 3-sulfate (DHEAS) and estrone 3-sulfate (E_1_3S), glucuronides such as estradiol 17-*β*-d-glucuronide (E_2_17*β*G), and the monoanionic bile acids glycocholate and taurocholate, as well as folic acid and its analog methotrexate [10, 11]. Interestingly, there is no putative mouse or rat orthologous gene corresponding to human* ABCC11* [12], indicating that* ABCC11* is not an orthologous gene but rather a paralogous gene generated by gene duplication within the human genome.

The SNP 538G>A in the* ABCC11* gene was originally identified as a determinant of human earwax type [13]. Human earwax, a secretory product of the ceruminous apocrine glands, is classified into wet and dry types. Both* ABCC11* 538G/G and 538G/A correspond to the wet type, and 538A/A corresponds to the dry type. The latter is the recessive phenotype and is commonly found within the Asian population (Korean, Chinese, and Japanese populations), whereas the former is the dominant phenotype and is found majorly in Africans and Caucasians. Accordingly, the frequency of the 538A allele is predominantly high among the Mongoloid populations in Asia but low among Africans and Caucasians. Thus, humans with the wet type of earwax naturally have a strong axillary odor, whereas those with dry earwax have little odor. Interestingly, this association had already been pointed out in the middle of the 20th century [14, 15]. In this context, wet earwax is regarded as an apocrine gland-related phenotype. The family history of axillary osmidrosis and its autosomal dominant pattern has also been recognized [16], and the wet type of earwax has been used as one of the phenotype-based diagnostic criteria for axillary osmidrosis [17].

## 2. A New Method for Genotype-Based Diagnosis

### 2.1. Rapid Genotyping of* ABCC11* 538G>A to Assess the Risk of Axillary Osmidrosis

**Figure 1:**
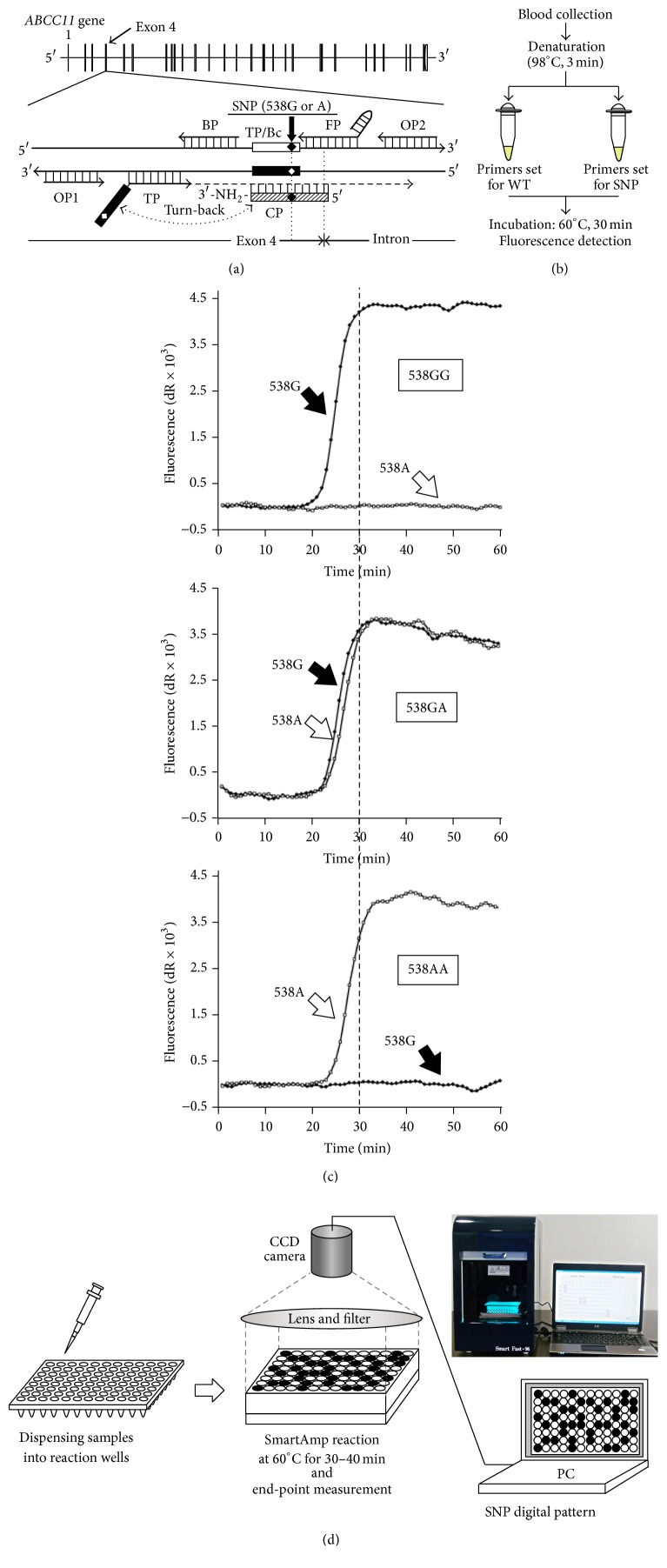
Genotyping at* ABCC11* 538G>A by the SmartAmp method. (a) Flowchart of the SmartAmp-based genotyping. After a simple heat treatment to degrade RNA and denature proteins, blood samples were added to the reaction mixture (total 25 *μ*L) and subjected to isothermal incubation at 60°C for 30 min while the fluorescence intensity was monitored. (b) Detection of the SNP 538G>A in* ABCC11* by the SmartAmp method. Time-dependent increases in fluorescence intensity produced by the SmartAmp reaction with* ABCC11* allele-specific primers carrying 538G (WT) or 538A (SNP) alleles were monitored by a real-time PCR system (Mx3000P; Stratagene). (c) Schematic illustration of the human* ABCC11* gene and relative positions of each primer for SmartAmp-based genotyping. The details of the DNA amplification process were described in Aw et al. [19]. (d) Schematic illustration for multiple end-point detection of SmartAmp-based SNP typing with a CCD camera-linked digital processor.

The association between the* ABCC11* genotype (538G>A) and axillary osmidrosis has enabled us to perform genotyping-based diagnosis of axillary osmidrosis, which is considered to be more objective than the phenotype-based diagnosis. Therefore, in the clinical setting, rapid, simple, and cost-effective methods are required for on-demand genotyping. To achieve this objective, we have recently developed a simple method targeting the SNP 538G>A in the human* ABCC11* gene based on an isothermal DNA amplification technique [18] (Figures 1(a)–1(c)). The new method enabled the determination of the genotype within 30 min under isothermal reaction conditions without necessitating the isolation of genomic DNA and sequential PCR steps [1, 2].

### 2.2. Genotyping Procedure

In the method we developed, the entire DNA amplification process is achieved by designing a total of five primers, namely, turn-back primer (TP), forward primer (FP), boost primer (BP), and outer primers 1 and 2 (OP1 and OP2) (Table 1). In addition, to inhibit the background amplification from mismatch sequence pairs, a competitive probe (CP) was constructed for effective genotyping at the SNP 538G>A in the* ABCC11* gene.

Since this method requires only a small volume (1-2 *μ*L) of peripheral blood, genotyping is easy [19]. Each SNP typing reaction is performed in a 25 *μ*L reaction mixture under an isothermal condition at 60°C. The mixture contains 2.0 *μ*M FP, 2.0 *μ*M TP, 1.0 *μ*M BP, 0.25 *μ*M each of the OPs, 20 *μ*M CP, 1.4 mM dNTPs, 5% DMSO, 20 mM Tris-HCl (pH 8.0), 10 mM KCl, 10 mM (NH_4_)_2_SO_4_, 8 mM MgSO_4_, 0.1% (v/v) Tween®20, 1/100,000 diluted SYBR® Green I (Takara Bio Inc., Shiga, Japan), and 0.24 U/*μ*L Aac DNA polymerase (K.K. DNAFORM, Yokohama, Japan). The polymorphism 538G>A is distinguished by TPs. DNA amplification and subsequent self-priming elongation (larger DNA production) are induced by the allele-specific TP and FP.

A multiple end-point determination of the SNP-dependent DNA amplification signal, an increase in the fluorescence of SYBR Green I, can be achieved by introducing a CCD camera and computational data acquisition, resulting in a simpler and more cost-effective detection (Figure 1(d)) [20].

### 2.3. DNA Amplification Process in the SmartAmp-Based Method

In the first step of the isothermal SmartAmp-based DNA amplification, FP and TP hybridize the template genomic DNA. Next, amplification products primed by each primer are detached from template genomic DNA. This process is induced by strand-displacing DNA polymerase, whose extensions are primed by OP1 and OP2, respectively. Subsequently, single-stranded amplification products become new templates in the second amplification step for opposing FP and TP. Due to the special features of the FP and TP, those amplicons will refold at their 3′ and 5′ ends to form new priming sites that maintain self-amplification in the further self-primed DNA elongation. The formation of concatenated DNA products in the SmartAmp reaction was schematically illustrated in our previous report [19].

The CPs inhibit the background amplification from mismatch sequence pairs. For example, the CP for the detection of WT (538G) allele is designed as a complementary sequence around the alternative (538A) allele and its 3′ end is modified by amination (Figure 1(a)). Therefore, this CP (538G) inhibits the misannealing of FP for WT allele and the following SmartAmp-based amplification from the genomic DNA carrying SNP (538A) allele. The CP (538A) enhances the assay specificity of allele-specific amplification in the similar manner.

### 2.4. Clinical Decision and Treatments of Axillary Osmidrosis

**Figure 2:**
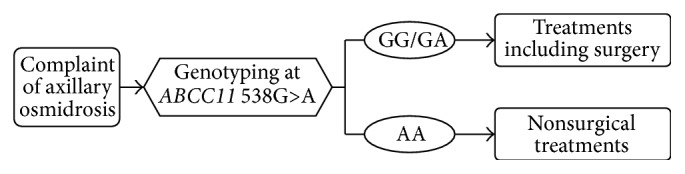
Gene-targeted strategy for the diagnosis and treatment of axillary osmidrosis. This strategy would be useful for the objective diagnosis of axillary osmidrosis, especially in ORS patients who have subjective olfactory delusion.

The genetic test for a SNP in the* ABCC11* gene is one of the clinical factors that underlies a doctor's decision. Patients carrying genotypes of 538G/G or 538G/A may be subjected to a surgical operation of excising apocrine glands, whereas such surgery is not indicated for those who are carrying the genotype of 538A/A (Figure 2).

Epidemiologic surveys indicate that subjects with* ABCC11* 538A/A have little risk of axillary osmidrosis, unlike 538GG/GA subjects. This SNP has been reported to correlate with deodorant usage, at least within the European population [21], while subjects with the 538A/A genotype are not directly affected by axillary osmidrosis. In particular, for the management of patients with olfactory reference syndrome (ORS) who tend to opt for aggressive surgical treatment simply due to the delusion of body odor, genetic evidence would be a powerful tool for diagnosis and nonsurgical treatment. Therefore, we proposed the clinical decision tree shown in Figure 2 [2].

### 2.5. Distinguishing Olfactory Reference Syndrome and Axillary Osmidrosis

The genotype-based diagnosis would be useful especially for olfactory reference syndrome (ORS) patients. Many ORS patients, also referred to as “jiko-syu-kyofu” in the Japanese medical literature, are characterized by having a preoccupation with the idea that their body emits a foul body odor that may be strong and/or offensive to others [22, 23]. These patients are convinced that they are the source of a strong smell, even if the people around them deny it. This kind of olfactory delusion, the main feature of ORS, is sometimes recognized by medical doctors during diagnosis of axillary osmidrosis. ORS patients tend to hope that surgical resection of the axillary apocrine glands will fundamentally resolve their problem, even if this is contrary to the clinical judgment of the doctor. Since the subjective diagnosis of this odor-producing disease by a doctor is psychologically difficult for these patients to accept, objective evidence indicating that they have no risk is important for dissuasion from surgery. Moreover, it would be unfortunate if the distress of body odor was not alleviated despite having undergone surgery, leading such patients to become even more nervous regarding their odor. Hence, an unnecessary surgery based solely on a request from patients or their family should be avoided, even if they are anxious about it. Therefore, genotyping the* ABCC11* SNP 538G>A can provide scientific evidence in an objective manner as an alternative approach to subjective/experienced assessment by clinicians.

## 3. More Insight into* ABCC11* 538G>A

**Figure 3:**
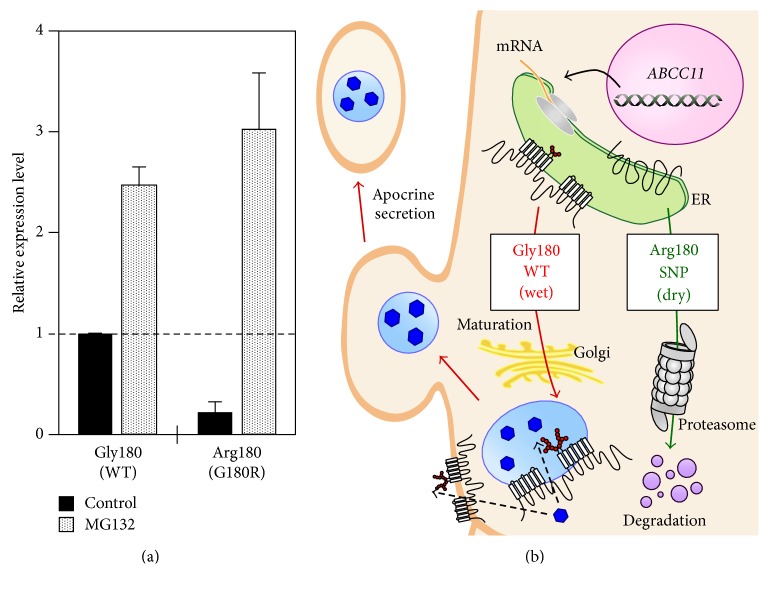
Effects of the SNP variant (Arg180) on the protein level and intracellular degradation of ABCC11. (a) To assess the effect of the Arg180 variant on the protein level of ABCC11, Flp-In-293 cells expressing the WT or Arg180 variant of ABCC11 were cultivated in the presence of MG132, a proteasome inhibitor, for 24 h. ABCC11 WT and Arg180 variant proteins were analyzed by immunoblotting with ABCC11-specific antibody after treatment with PNGase F, a glycosidase. The signal intensity ratio (ABCC11/GAPDH, internal control) was normalized to the control and expressed as mean ± SD. (b) Schematic illustration of the posttranslation modification of ABCC11 WT and proteasomal degradation of the Arg180 variant.

While more than 10 nonsynonymous SNPs are found in the human* ABCC11* gene, only 538G>A (Gly180Arg) is directly related to the phenotypes above described. The SNP 538G>A is located in exon 4 where Gly180 is substituted by Arg180 in the putative first transmembrane domain of the ABCC11 protein. This amino acid substitution results in constitutional instability of the nascent ABCC11 protein. The SNP variant ABCC11 Arg180 undergoes proteasomal degradation and loses its intracellular function (Figure 3). This molecular mechanism is consistent with the fact that apocrine gland-related phenotypes such as human earwax and axillary osmidrosis are Mendelian traits [1]. In fact, fluorescence immunohistochemistry analyses of the human apocrine gland show that ABCC11 Gly180 (wild type: WT) is expressed in the gland, and the nonsynonymous SNP 538G>A greatly affects the cellular localization of the ABCC11 protein in apocrine secretory cells [1].

**Figure 4:**
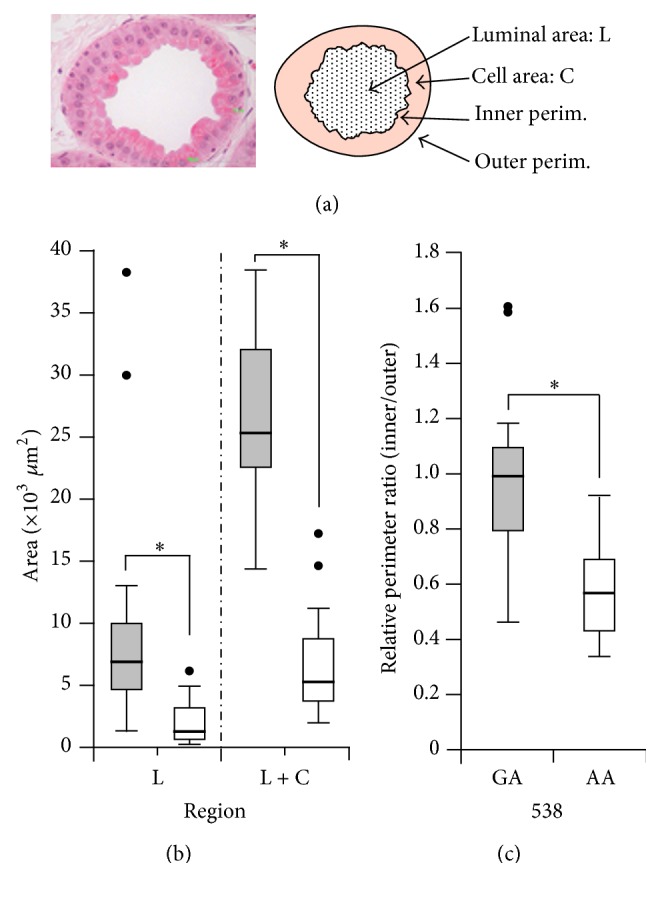
Image analysis of human apocrine glands with ABCC11 538GA and 538AA. (a) Typical image of human apocrine glands in the external auditory canal (left). Schematic illustration of measurement parameters for image analysis (right). (b and c) Well-developed apocrine glands in a subject with* ABCC11* 538GA (grey) as compared with a subject with 538AA (white). Histological images of human apocrine glands [1] were analyzed by the ImageJ program (v1.46d). Calculated data are expressed as box plots. Differences were considered significant when *p* < 0.01 (^*∗*^).

Although the endogenous substrate of ABCC11 determining the dominant phenotype has not been elucidated to date, recent findings suggest that ABCC11 WT contributes to development and/or regulation of the secretion activity of human apocrine glands [24, 25]. For instance, during surgical resection, large and extensive apocrine glands are usually observed in the axillae of patients with axillary osmidrosis [26]. The wet type of earwax is derived from the secretion product of the apocrine gland in the external auditory canal, whereas this apocrine secretion is lacking in the dry-type phenotype. Furthermore, according to our preliminary analyses of tissue sections from ceruminous apocrine glands, the luminal area of the apocrine glands of a 538G/A subject was larger than that of a 538A/A subject (Figure 4). Since the total secretion activity of apocrine glands depends on tissue development and intracellular mechanisms regulating apocrine secretion, ABCC11 WT would be responsible for the development of human apocrine glands, resulting in the excess production of apocrine sweat, including the precursor compounds of axillary odor. Recently, Harker et al. demonstrated that the* ABCC11* 538A/A genotype did not result in the complete absence of precursors and produced significantly lower levels of precursor compounds as compared with the 538G/G or G/A genotype [27]. These findings suggest that apocrine glands with the* ABCC11* 538A/A genotype have little secretory activity. They surmised that their results were due to the extremely low level of ABCC11 activity in the Arg180 variant. A more rational explanation of this minimal secretion, however, might be that other contributors regulating the total secretory activity of apocrine glands besides ABCC11 also contribute to the promotion of apocrine secretion. To clarify this, we need to further address the issue of how ABCC11 proteins are involved in the regulation of apocrine glands.

**Figure 5:**
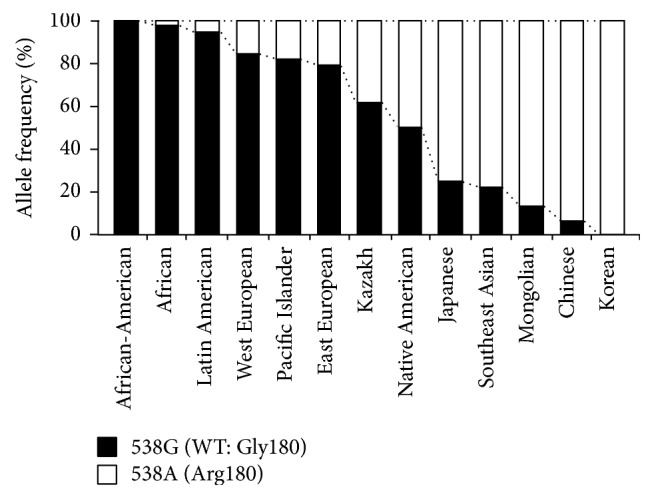
Allele frequencies of* ABCC11* 538G (WT; Gly180) and 538A (Arg180) among different ethnic populations. Data are calculated from Yoshiura et al. [13].

Interestingly, as shown in Figure 5, the allele frequency of the SNP 538G>A in the human* ABCC11* gene exhibits wide differences across different ethnic groups [13], reflecting the genetic diversity of the human genome that occurred during the history of intercontinental migrations of* Homo sapiens* [9, 24]. According to the popular theory of* out of Africa*, ancient humans should have had the* ABCC11* 538G allele which corresponds to the high secretory phenotype of apocrine glands. Considering the production of pheromone-like compounds in human armpits probably originating from their axillary apocrine glands [17, 28], axillary odor directly or indirectly regulated by ABCC11 might have been important for nonverbal communication among ancient* Homo sapiens* and our ancestors.

## 4. Nature of Body Odor

### 4.1. Characteristics of Axillary Osmidrosis

Humans tend to emit peculiar body odors, but each individual might have a chronic body odor or little odor. Across most cultures, body odor is often perceived as being unpleasant by other persons, whereas it also affects the confidence and self-esteem of the person emitting the odor. To address this problem, mankind has developed ways and means to manage body odor, for example, by the use of deodorants, refreshing sprays, and perfumes. In modern society, elimination of body odor is part of daily grooming, like hand-washing, hair styling, and other similar activities. Nevertheless, the genetic and/or environmental factors that give rise to individual differences in body odor and the molecular mechanisms thereof remain unclear.

Axillary osmidrosis is a condition characterized by strong odors and profuse sweating from the axillae [29]. The symptoms of axillary osmidrosis generally develop around the time of puberty when the apocrine glands existing from birth become active for the first time [30]. The yellow staining in the armpits of clothing also impacts the affected individual's quality of life. Thus, axillary osmidrosis is often perceived as an undesired problem, particularly by young women. Especially in Asian countries where persons with strong body odor comprise a minor population, axillary osmidrosis tends to be even more strongly disliked. In particular, it is true that axillary osmidrosis is recognized as a disease in Japan, and its clinical treatments are covered by the national health insurance system.

**Figure 6:**
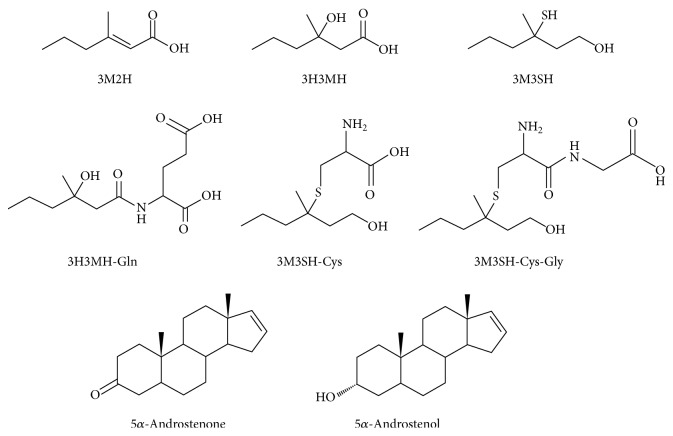
Chemical structures of compounds underlying human axillary odor.

The major contributors to axillary odor are unsaturated fatty acids, hydroxylated branched fatty acids, sulfanylalkanols, and some steroids represented by (E)-3-methyl-2-hexenoic acid (3M2H) [31], 3-hydroxy-3-methyl-hexanoic acid (3H3MH), 3-methyl-3-sulfanylhexan-1-ol (3M3SH) [32, 33], 5*α*-androstenone, and 5*α*-androst-16-en-3*α*-ol [34, 35], respectively (Figure 6). In addition, an* in vitro* transport experiment showed that the glutathione conjugate of 3M3SH, a putative precursor of 3M3SH, was transported by ABCC11 WT [36], suggesting the involvement of ABCC11 in the formation of axillary odor. Some precursors of those odorous components have been found to be secreted from axillary apocrine glands [5, 37, 38], which suggests that the inhibition of secretion and/or development of axillary apocrine glands would contribute to the prevention or treatment of axillary osmidrosis.

## 5. Future Perspectives

Apart from axillary osmidrosis,* ABCC11* genotypes might be associated with the risk of breast cancer or drug-induced toxicity [25, 39–41]. According to recent studies, women in the Japanese population with the 538G allele in* ABCC11* had a higher risk of breast cancer than those with the 538A allele [41], whereas this association was not found in the Caucasian population [42, 43]. It has also been shown by a Japanese research group that the expression of ABCC11 in women with breast cancer is associated with aggressive phenotypes and poor disease-free survival [44]. Since some anticancer agents and metabolites thereof are substrates of ABCC11, a patient's response to nucleoside-based chemotherapy could be affected by the* ABCC11* genotype [25]. A recent Caucasian human liver cohort study has shown an association between* ABCC11* SNP 1637C>T (Trp546Met) and the risk of toxicity of 5-fluorouracil, which is a widely used antipyrimidine anticancer drug [40]. There is no finding, however, on the relationship between the* ABCC11* 538A allele and the risk of toxicity of 5-fluorouracil in this European case study. Further studies, preferably in Asia, where the 538A allele is predominant, are warranted to shed light on this question. In addition, it remains to be clarified how ABCC11 affects breast cancer or its origin. Interestingly, an evolutionary derivation of mammary glands from apocrine glands has been suggested based on their biological similarities [45]. Since the functional form of ABCC11 might contribute to the development and/or activity control of both mammary glands, as well as apocrine glands, further verifications would be required from various aspects including not only chemoresistance but also the physiological function and regulation of ABCC11.

## 6. Conclusions

In this review article, we have addressed the clinical importance of human* ABCC11* 538G>A as a risk factor of axillary osmidrosis by using an efficient genotyping method. This genotyping-based strategy would be helpful in making the diagnosis of axillary osmidrosis, since the objective evidence acquired would relieve ORS patients of their odor delusion. Although the molecular mechanisms regulating the secretory process in apocrine glands have not been clearly understood, accumulating evidence strongly suggests that ABCC11 contributes to the function of apocrine glands and* ABCC11* 538G>A reflects their activity. Further studies are needed to elucidate the physiological function of ABCC11 and its endogenous substrates secreted from apocrine glands. Therefore, the validation of clinically relevant genetic factors and the development of systems to be used for personalized medicine would be the next important steps.

## Figures and Tables

**Table 1:** Primers sets for the detection of WT and SNP alleles in the human *ABCC11* gene.

WT (538G) detection primers set (5′to 3′)	
TP	**C**GAGTACACTGGTTGATTTTCGATGCACTTC
FP	agcgatgcgttcgagcatcgctGTCTGCCACTTACTGGCC
BP	AGAAGCAGATGCCCAGAA
OP1	TGATGCTGAGGTTCCAG
OP2	TAGAGTCCCCCAAACCT
CP	TACTGGCC**T**GAGTACAC-NH_2_

SNP (538A) detection primers set (5′to 3′)	

TP	C**T**GAGTACACTGGTTGATTTTCGATGCACTTC
FP	agcgatgcgttcgagcatcgctGTCTGCCACTTACTGGCC
BP	AGAAGCAGATGCCCAGAA
OP1	TGATGCTGAGGTTCCAG
OP2	TAGAGTCCCCCAAACCT
CP	TACTGGCC**C**GAGTACAC-NH_2_

TP: turn-back primer; FP: forward primer; BP: boost primer; OP: outer primer; CP: competitive probe.
